# Burden of Oral Cancer on the 10 Most Populous Countries from 1990 to 2019: Estimates from the Global Burden of Disease Study 2019

**DOI:** 10.3390/ijerph19020875

**Published:** 2022-01-13

**Authors:** Shu-Zhen Zhang, Long Xie, Zheng-Jun Shang

**Affiliations:** 1 The State Key Laboratory Breeding Base of Basic Science of Stomatology (Hubei-MOST) & Key Laboratory of Oral Biomedicine Ministry of Education, School & Hospital of Stomatology, Wuhan University, Wuhan 430079, China; zhangshuzhen@whu.edu.cn (S.-Z.Z.); xielong@whu.edu.cn (L.X.); 2 Department of Oral and Maxillofacial-Head and Neck Oncology, School and Hospital of Stomatology, Wuhan University, 237 Luoyu Road, Wuhan 430079, China

**Keywords:** oral cancer, incidence, death, disability-adjusted life years, global burden of disease

## Abstract

Background: Oral cancer (OC) is a common tumour that poses a threat to human health and imposes a heavy burden on countries. This study assessed the burden imposed by OC on the 10 most populous countries from 1990 to 2019 on the basis of gender, age and socio-demographic index. Methods: Data on incidence, mortality, disability-adjusted life years (DALY) and corresponding age-standardised rates (ASR) for OC in the 10 most populous countries from 1990 to 2019 were derived from the Global Burden of Disease Study 2019. Estimated annual percentage changes were calculated to assess the trends of morbidity, mortality and DALY. The indicator that served as a proxy for survival rate was the supplement of mortality-to-incidence ratio (SMIR) (1 − (M/I)). Results: The number of new cases, deaths and DALY have increased in all 10 countries in the past 30 years. Trends in age-standardised incidence rates (ASIR), age-standardised mortality rate (ASMR) and age-standardised DALY for OC in the 10 most populous countries varied. The SMIR increased in all countries, with most countries having an SMIR between 30% and 50%. In 2019, the United States had the highest SMIR at 76%, whereas Russia had the lowest at 21.7%. Incidence and mortality were close between male and female subjects in Japan, Indonesia, Mexico, India, Bangladesh and Pakistan. The incidence and mortality in male subjects in the United States, Russia, China and Brazil were two or more times those of female subjects. Gender difference was highest among patients aged 40–69 years. Conclusion: Trends and gender differences in ASIR, ASMR and age-standardised DALY for OC vary in the 10 most populous countries. Government cancer programs are often expensive to run, especially in countries with large populations. Policy makers need to take these differences into account when formulating policies.

## 1. Introduction

Oral cancer (OC) is a malignant cancer occurring in the oral cavity. This type of cancer is a global public health concern with major socio-economic implications, but these implications are often overlooked in public health policy making. Globally, 354,864 new cases of OC and 177,384 OC-related deaths were recorded in 2018 [1]. The use of tobacco and smokeless tobacco (SLT), alcohol consumption and human papillomavirus (HPV) infection are major risk factors for OC [2,3,4]. Squamous cell carcinomas in the oral and oropharyngeal regions account for over 90% of malignancies. The standard treatment is surgery followed by radiation or chemotherapy. Five-year survival rates vary from 40% to 50% due to recurrence and secondary metastasis to cervical lymph nodes [5,6].

The incidence, prevalence and distribution of OC vary across regions of the world. The 10 most populous countries in the world are China, India, the United States, Indonesia, Brazil, Pakistan, Bangladesh, Russia, Japan and Mexico, which are distributed in Asia, North America, South America and Europe; these countries account for 56.3% of the total world population. India, Pakistan and Bangladesh are also the countries most affected by OC. Although the incidence of OC is not high in the world’s most populous country (i.e., China), the burden of OC is heavy due to China’s large population [7]. The economic cost of treating OC is high, placing a heavy burden on families and healthcare systems and having an adverse impact on the economies of countries, especially those with large populations [8]. Governments may not be able to effectively develop public health policies for their populations because of insufficient data on trends in morbidity, mortality, survival rate and disability-adjusted life years (DALY) for the disease for similar populations or economic levels.

The Global Burden of Disease Study 2019 (GBD 2019), coordinated with the Institute for Health Indicators and Assessment, estimated the burden of disease, injury and risk factors in 204 countries and regions and chose from numerous locations, and allowed students, researchers, policy makers and the public to access, view and interact with the GBD 2019 data output of other members. The GBD 2019 helps national policy makers correctly assess the burden of OC and rationally allocate limited health care system resources. A comparative study on the incidence, mortality, survival rate, spatial distribution and long-term trends for OC in the 10 most populous countries in the world has not been conducted. The aim of the present study was to reveal the incidence, mortality, survival rate and DALY of OC by sex, age and country in the 10 most populous countries and analyse their trends and causes.

## 2. Methods

### 2.1. Study Data

Data about the burden imposed by OC on the 10 countries with the largest populations were obtained from an online data source tool called the Global Health Data Exchange (GHDx) query tool [9]. This query tool is the product of an ongoing global collaboration and uses all available epidemiological data for the comparative assessment of health loss due to 364 diseases across 204 countries and territories. Furthermore, gender and age information were used to assess the effect of age and gender on the burden of OC. However, individuals that were younger than 5 years old were excluded because of insufficient data. The GBD 2019 provides data on the annual morbidity and mortality, DALY and respective age-standardised rates (ASRs) for OC from 1990 to 2019. Survival estimates are being used to monitor disease invasiveness, therapeutic efficacy and the burden of regular check-ups on the healthcare system. The indicator that served as a proxy for survival rate was the supplement of mortality-to-incidence ratio (SMIR) (1 − (M/I)) [10]. Data on tobacco smoking and chewing for the 10 countries were obtained from the GBD 2019 database. Data on alcohol consumption were obtained from the World Health Organization (WHO) Global Health Observatory [11].

### 2.2. Statistical Analysis

The annual age-standardised incidence rate (ASIR), age-standardised mortality rate (ASMR), SMIR, age-standardised DALY and estimated annual percentage changes (EAPCs) were calculated and used to assess the trends of the incidence, mortality, and DALY of OC. DALY were calculated by obtaining the sum of the years lived with disability and the life years lost [12]. ASR was obtained using the GHDx query tool and used as an objective indicator for quantifying the trend in cancer incidence. SMIR complements the mortality-to-incidence (M/I) ratio (1 − (M/I)). This ratio is a synonymous supplement to mortality, which is defined as the complement of the ratio of the number of deaths to the number of new cases of the same disease in the same period [10]. EAPC was used to describe the ASR’s trend over a specific time interval [13]. The natural logarithm of ASR has a linear relationship with time; hence, Y = α + βX + ε, where Y is ln (ASR), X is calendar year and ε is the error term. In this formula, β indicates the positive or negative trend in ASR. EAPC was calculated as follows: EAPC = 100 × (exp (β) − 1). The formula of EAPC and its 95% confidence interval (CI) were obtained using a linear model. When the lower limit of CI and the EAPC were positive, ASR was considered to have an upward trend. Conversely, when the upper limit of the CI and the EAPC were negative, the ASR was considered to have a downward trend. The relationship between ASR and socio-demographic index (SDI) in 2019 in different countries was also assessed to determine potential factors influencing ASR. Statistical Package for Social Sciences version 25.00 (IBM, Armonk, NY, USA) was used in the statistical analysis, and the significance level was set at a *p* value of < 0.05.

## 3. Results

### 3.1. Analysis of OC Incidence in the 10 Most Populous Countries

The number of OC cases increased in all countries over the past 30 years. The number of OC cases in China increased at most from 12,390.24 (95% UI, 10,867.31–14,056.85) in 1990 to 45,216.41 (95% UI, 37,690.41–54,179.36) in 2019, with an increase of 264.94%. Russia had the smallest increase, up by 38.48% from 5321.7 (95% UI, 5158.93–5530.02) in 1990 to 7369.72 (95% UI, 6377.52–8485.26) in 2019 (Table 1). ASIR exhibited a downward trend in Bangladesh, Brazil, Mexico and the United States; a stable trend in Russia; and an upward trend in five countries (i.e., China, India, Indonesia, Japan and Pakistan). Among the female subjects, the ASIR showed a downward trend in China and the United States, a stable trend in Bangladesh and Brazil, and an upward trend in the remaining six countries (i.e., India, Indonesia, Pakistan, Russia, Japan and Mexico). Among the male subjects, the ASIR in China, India, Indonesia, Pakistan and Japan showed a downward trend and exhibited an upward trend in the remaining five countries. The country with the fastest increase in ASIR was China, with an EAPC of 2.33 (95% CI, from 2.02 to 2.64), and the country with the largest reduction was Bangladesh, with an EAPC of −1.20 (from −1.36 to −1.05, 95% CI) (Figure S1A, Table 1, Table S1). The countries with the highest ASIR were Pakistan (21.93/100,000), India (8.82/100,000) and Bangladesh (6.12/100,000), and the countries with the lowest incidence rates were Mexico (1.63/100,000), Japan (2.24/100,000) and China (2.25/100,000) (Table 1).

**Figure 1:**
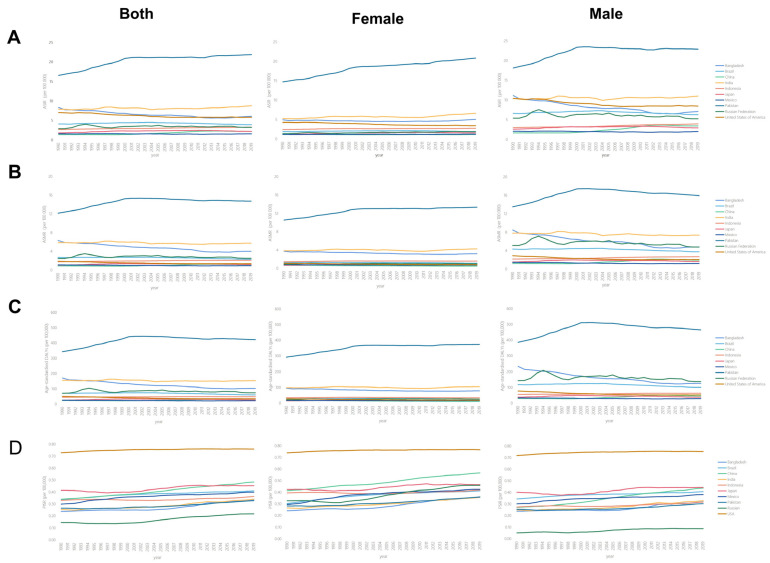
The change trends of age-standardized incidence, mortality, DALY rate and proxy indicator for survival rate among different countries. (**A**) Age-standardized incidence rate. (**B**) Age-standardized mortality rate. (**C**) Age-standardized DALY rate. (**D**) Supplement of mortality-to-incidence ratio. (Countries are ranked from highest to lowest in SDI.)

**Figure 2:**
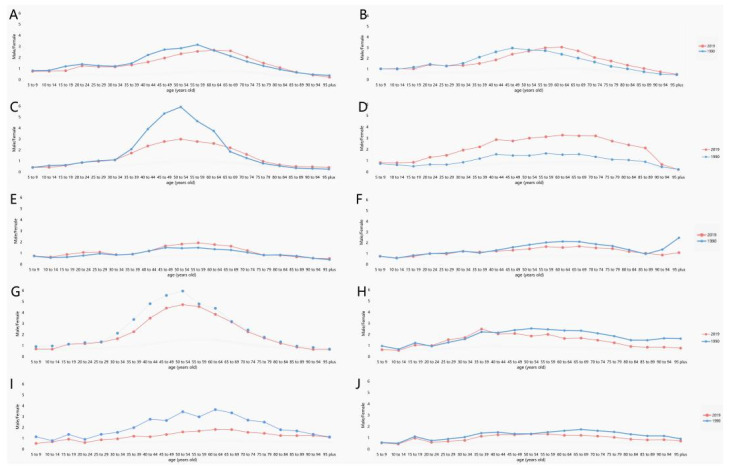
The ratio of male to female incidence of OC among different age groups in 1990 and 2019. (**A**) Japan, (**B**) USA, (**C**) Russian, (**D**) China, (**E**) Indonesia, (**F**) Mexico, (**G**) Brazil, (**H**) India, (**I**) Bangladesh, (**J**) Pakistan. (Countries are ranked from highest to lowest in SDI.)

**Figure 3:**
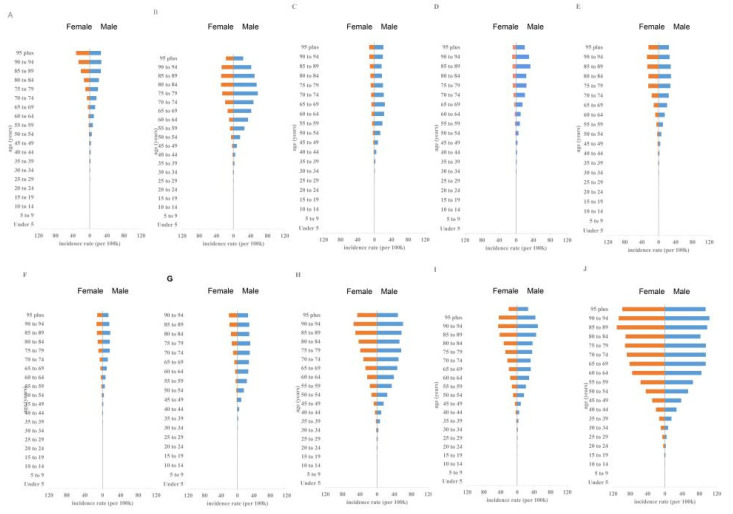
The incidence rates of OC in different age groups in 2019. (**A**) Japan, (**B**) USA, (**C**) Russian, (**D**) China, (**E**) Indonesia, (**F**) Mexico, (**G**) Brazil, (**H**) India, (**I**) Bangladesh, (**J**) Pakistan. (Countries are ranked from highest to lowest in SDI.).

Over the past 30 years, male subjects had higher ASIR than female subjects (Figure 1A). The male–female incidence ratio among different ages showed a unimodal distribution with a peak among peopled aged 50–54 years (Figure 2). ASIR tended to increase gradually with age. In 2019, incidence had a unimodal distribution and increased among people aged 50+ years (Figure 3).

**Figure 4:**
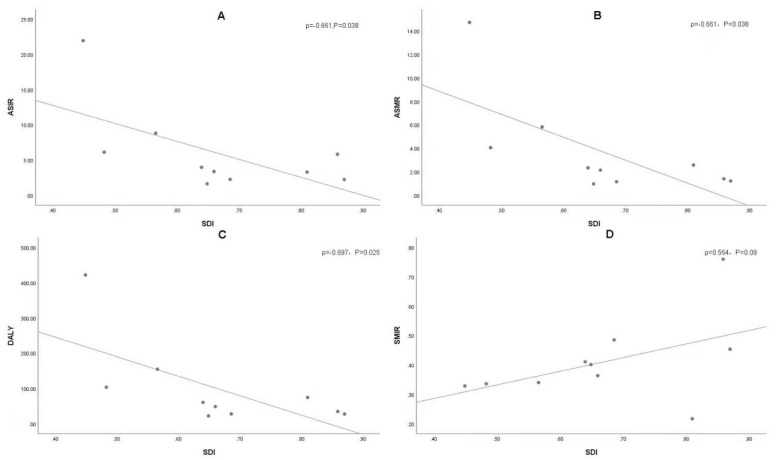
The correlation between SDI and oral cancer ASR (incidence (**A**), death (**B**), DALY (**C**) and SMIR (**D**)) in 2019.

**Figure 5:**
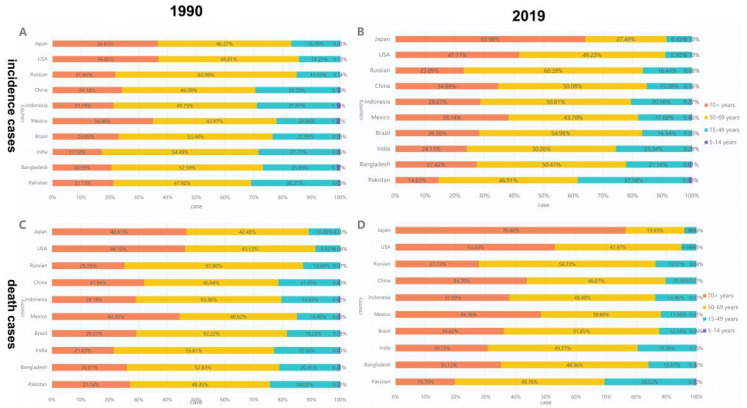
Distribution of different ages in oral cancer incidence/death cases by country. (**A**) Incidence in 1990. (**B**) Incidence in 2019. (**C**) Death rate in 1990. (**D**) Death rate in 2019. (Countries are ranked from highest to lowest in SDI.)

A negative correlation was observed between ASIR and the 2019 SDI (ρ = −0.661, *p* = 0.038) (Figure 4A). Results revealed that the higher the SDI, the higher the proportion of incidence cases among the elderly out of all OC incidence cases (Figure 5). From 1990 to 2019, the proportion of the incidence cases among young people decreased annually and that of the elderly increased yearly in eight countries (i.e., Japan, USA, China, Indonesia, Mexico, Brazil, India and Bangladesh). As shown in Figure S2, the situation in Pakistan was opposite with those of most countries, wherein the proportion of incidents among the young increased; the proportion of annual incidence cases among the young and elderly in Russia remained stable.

### 3.2. Analysis of OC-Related Death in the 10 Most Populous Countries

The number of deaths increased in all countries over the past 30 years, with the fastest increase observed in China (205.84%) and Russia having the slowest increase (30.00%) (Table 2). The ASMR showed a downward trend in Bangladesh, Brazil, India, Mexico and the United States; a stable trend in Russia and Japan; and an upward trend in China, Indonesia and Pakistan. Among female subjects, the ASMR showed a downward trend in Bangladesh, Brazil, China, Mexico and the United States; a stable trend in India and Russia; and an upward trend in Indonesia, Pakistan and Japan. Among male subjects, the ASMR showed a downward trend in Bangladesh, Brazil, India, Mexico, Russia and the United States; a stable trend in Japan; and an upward trend in China, Indonesia and Pakistan. The ASMR among Chinese men increased significantly, with an EAPC of 2.56 (from 2.17 to 2.96, 95% CI), while the ASMR decreased significantly in Bangladeshi men, with an EAPC of −2.06 (from −2.23 to −1.88, 95% CI) (Figure S1B, Table 2, Table S2). The countries with the highest ASMR in 2019 were Pakistan (14.72), India (5.81) and Bangladesh (4.06), while the countries with the lowest ASMR were Mexico (0.98), China (1.16) and Japan (1.22) (Table 2).

Over the past 30 years, male subjects had higher ASMR than female subjects (Figure 1B). The male-to-female death ratio across different ages showed a unimodal distribution in most countries in 2019 (Figure S3). The ASMR tended to increase gradually with age. The death rate among people aged 70+ years showed a unimodal distribution and increased in 2019 (Figure S4).

The ASMR and SDI in 2019 were found to be significantly correlated (ρ = −0.661, *p* = 0.038) (Figure 4B). From 1990 to 2019, the death toll among people aged 70+ years in the United States, Mexico, Japan, Indonesia, India, China, Brazil and Bangladesh increased, whereas the death toll of people aged < 14 years decreased, especially in Japan, where the death toll of people aged 75+ years increased from 46.6% in 1990 to 76.5% in 2019. The death rates in Russia remained stable, whereas the death rate among people aged 70+ years in Pakistan decreased significantly, from 27% in 1990 to 19.7% in 2019 (Figure 5, Figure S5).

### 3.3. Analysis of DALY and Survival Rate for OC in the 10 Countries with the Largest Populations

DALY has increased in all countries in the past 30 years, with the fastest growth observed in China (158.62%) and the slowest growth in the United States (20.43%). The 10 countries with the highest DALY in 2019 were India (1,922,663.79), Pakistan (594,088.79) and China (575,805.4), whereas Mexico (27,215.25), Japan (77,039.22) and India (113,143.08) had the lowest DALY. The countries with the highest ASR of DALY were Pakistan (421.87), India (154.9) and Bangladesh (103.95), whereas the countries with the lowest ASR of DALY were Mexico (22.56), Japan (27.73) and China (28.27). The DALY of China, Indonesia and Pakistan increased; those of the United States, Brazil, Russia, Bangladesh, Mexico and India decreased; and that of Japan remained stable. The largest increase in DALY was observed in China, with an EAPC of 1.24 (from 0.96 to 1.52, 95% CI), whereas the largest decrease was in Bangladesh, with an EAPC of −1.81 (from −1.93 to −1.68, 95% CI) (Table 3, Table S3, Figure S1C).

Over the past 30 years, male subjects had higher age-standardised DALY than female subjects (Figure 1C). The ratios of male-to-female DALY across different ages exhibited a unimodal distribution in most countries in 2019, with a peak among people aged 60–64 years (Figures S6 and S7).

Age-standardised DALY and SDI in 2019 were found to be negatively correlated (ρ = −0.697, *p* = 0.025) (Figure 4C). In addition, no significant correlation was found between SMIR and SDI in 2019 (ρ = 0.564, *p* = 0.09) (Figure 4D).

OC SMIR increased in all countries from 1990 to 2019, with the fastest increase occurring in China (from 0.34 to 0.48). In 2019, the highest OC SMIR was found in the United States (0.76), China (0.48) and Japan (0.45), whereas the lowest OC SMIR was found in Russia (0.22), Pakistan (0.33) and Bangladesh (0.34), among which the lowest was found among Russian men, with a SMIR of only 0.09 (Figure S8).

### 3.4. Etiological Analysis

**Figure 6:**
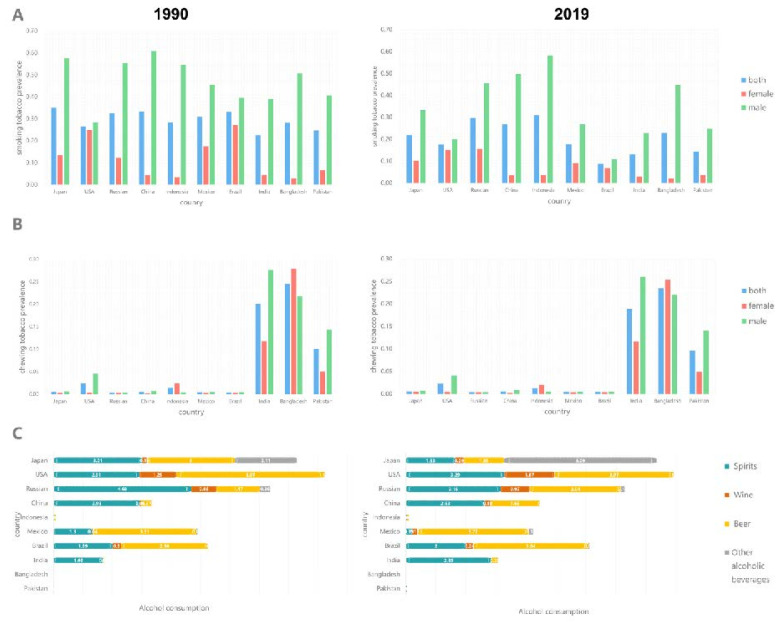
The prevalence of smoking tobacco and chewing tobacco and the consumption of alcohol in ten countries in 1990 and 2019. (**A**) Smoking tobacco prevalence in 1990 and 2019; (**B**) Chewing tobacco prevalence in 1990 and 2019; (**C**) Alcohol, recorded per capita (15+) consumption (in litres of pure alcohol) in 1990 and 2019.

The prevalence of tobacco smoking decreased in nine countries (except Indonesia) from 1990 to 2019, with Brazil showing the fastest decline. In 2019, Indonesia, Russia and China had the highest tobacco smoking prevalence, whereas Brazil, India and Pakistan had the lowest rates (Figure 6A).

Tobacco chewing prevalence has distinct regional characteristics, with high prevalence in the South Asian countries of Bangladesh, India and Pakistan, where the prevalence of tobacco chewing has barely changed from 1990 to 2019. The tobacco chewing prevalence in the remaining seven countries was extremely low (Figure 6B).

Recorded per capita consumption of pure alcohol (over 15 years of age) was calculated as the sum of specific consumption of pure alcoholic beverages (e.g., beer, wine, spirits) in litres of pure alcohol. Bangladesh, Pakistan and Indonesia do not consume alcohol. From 1990 to 2019, per capita alcohol consumption in Japan, India, China and Brazil increased, whereas reductions were observed in the United States, Russia and Mexico. In 2019, the countries with the highest per capita alcohol consumption were the United States (8.93 L), Japan (8.36 L) and Russia (7.29 L), and the countries with the highest spirits consumption were the United States (3.29 L), Russia (3.16 L) and India (2.85 L) (Figure 6C).

## 4. Discussion

This study is the first to analyse the latest trends and patterns in OC incidence, mortality, survival and DALY in the 10 countries with the largest populations from 1990 to 2019 on the basis of the GBD 2019 database. The number of cases, deaths and DALY generally increased in all countries. Compared with global or state studies, the present study has a small amount of data, which can reduce systematic errors in data analysis and improve the accuracy of the results. Moreover, the 10 countries we studied were highly representative, because they accounted for 56.3% of the total population of the world, including the highest incidence rate in South Asian countries and the lowest incidence rate in East Asia.

Overall, the morbidity and mortality rates are significantly high in South Asian countries such as Pakistan, India and Bangladesh, followed by the United States, Brazil, Indonesia and Russia, and low in China, Japan and Mexico. The major risk factors for OC are the use of tobacco and SLT and alcohol consumption. SLT refers to various tobacco products, including snuff, DIP (dipped tobacco or wet snuff) and smokeless chewing tobacco. The three countries with the highest tobacco chewing prevalence, moderate tobacco smoking prevalence and lowest alcohol consumption (i.e., Pakistan, India and Bangladesh) also had the highest rates of OC incidence, mortality and DALY in 1990 and 2019, revealing a strong relationship between tobacco chewing and OC. Among India (0.19), Pakistan (0.1) and Bangladesh (0.24), Pakistan surprisingly has the lowest chewing tobacco prevalence but the highest incidence, mortality and DALY for OC. This condition may be due to the type, amount, frequency and duration of tobacco chewing most commonly used in Pakistan. The most commonly used tobacco chewing products in Bangladesh, India and Pakistan are Zarda, Khaini and Naswar, respectively [14]. Among the tobacco chewing products used in South Asia, Naswar is one of the tobaccos with the highest specific nitrosamine content and alkalinity [15]. Figure 6 shows that the United States, Brazil and Russia have high per capita alcohol consumption and high tobacco smoking prevalence; these countries also have high ASIR and ASMR for OC. Indonesia has a low alcohol consumption rate; the high incidence rate for OC in Indonesia is due to the high prevalence of tobacco smoking and chewing. Although the ASIR and ASMR in China and Japan were relatively low, tobacco smoking prevalence and alcohol consumption were relatively high, and both the ASIR and ASMR increased. Mexico had the lowest ASIR and ASMR among the 10 countries in 2019 and exhibited a downward trend, in line with trends in smoking prevalence and alcohol consumption. From 1990 to 2019, the change trend of ASIR and ASMR was fundamentally consistent with that of tobacco smoking and chewing prevalence and alcohol consumption. The WHO’s MPOWER framework has reduced tobacco smoking prevalence in most countries (except Indonesia) since its implementation. Brazil exhibited the fastest decline, because Brazil is one of the two countries in the world to have fully implemented all MPOWER measures [16]. In Indonesia, no significant progress has been made except for the introduction of a 40% pictorial health warning on packaging. Indonesia remains one of the few countries to advertise cigarettes on television, and cigarette taxes are among the lowest in the world [17]. In addition, the prevalence of tobacco chewing remains unchanged, while alcohol consumption is increasing. Considering their significant influence on OC incidence, governments should strengthen control over the prevalence of tobacco chewing and alcohol consumption to reduce the burden of OC.

In 2019, the gender difference in China was the largest among the 10 countries, with ASIR among men being 2.85 times that of women and ASMR being 3.5 times that of women. This finding can be explained by considerable differences in the tobacco smoking prevalence and alcohol consumption between men and women in China. The tobacco smoking prevalence in China in 2019 was 27%, with 50% among males and only 4% among females (Figure 6A). The alcohol consumption rate among Chinese adults in 2018 was 30.5%, with 53.8% among males and 12.2% among females [18]. Other countries with high ratios of male-to-female ASIR and ASMR, including Brazil, Russia and the United States, have similar situations [19,20,21]. In India, Pakistan, Bangladesh and Indonesia, where SLT use is prevalent, the ratios of male-to-female ASIR and ASMR are low because of the high tobacco chewing prevalence among women and the significantly high OC risk associated with tobacco chewing among women compared with that of men [22].

Over the past 30 years, the morbidity and death rates among young people in the United States, Mexico, Japan, Indonesia, India, China, Brazil and Bangladesh have declined, which may be related to economic development, population growth and aging. Especially in Japan, the proportion of incidence cases among people aged 70+ years increased from 36.6% in 1990 to 64% in 2019, and the number of deaths increased from 46.6% in 1990 to 76.5% in 2019 (Figure 5). This condition can be explained by improved healthcare or appropriate prevention [23], population aging and low fertility rate. However, the proportion of people aged 70+ years in Pakistan decreased, whereas that of people aged 15–49 years increased due to increasing SLT use among teenagers [24]. The proportion of patients over 75 years of age has dropped from 21.1% in 1990 to 14.6% in 2019, and the number of deaths dropped from 27% in 1990 to 19.7% in 2019 (Figure 5).

SDI negatively correlated with ASIR, ASMR and age-standardised DALY in 2019. The risk factors for OC in high SDI countries are mainly tobacco and alcohol consumption, whereas the main risk factor for OC in low SDI countries is tobacco chewing (Figure 6). Each country should, therefore, take appropriate measures to reduce the burden of OC according to its economic situation, and the main causes of OC. OC survival was not significantly associated with SDI, especially in Russia. As a country with high SDI, Russia had the lowest survival rates due to delayed treatment, late diagnosis and poor ability of modern methods [25]. Russia has the most dangerous pattern of alcohol abuse and the highest per capita alcohol consumption in the European region and is classified as ‘most at risk’ by the WHO [26,27,28]. Russia’s medical system, a legacy of the Soviet Union, hampers the international exchange of medical knowledge. Graduate and post-graduate education in oncology and pathology (and general medical education) in Russia is also severely hampered by the lack of up-to-date medical literature and journals [29,30,31,32,33]. China and Indonesia are middle SDI countries, and their rapidly growing burden of oral cancer is alarming. Additionally, as a middle SDI country, with the implementation of strong tobacco control measures in Brazil, the burden of oral cancer has been reduced, which is worth learning. As the lowest SDI countries, Pakistan, India and Bangladesh have the highest ASIR, ASMR and DALY of oral cancer. Tobacco control may be the most cost-effective solution to reduce the burden of oral cancer.

The overall five-year survival rate for OC of nearly 50% is the lowest worldwide. Despite recent advances in treatment, prognosis among patients with OC remains poor [34]. The United States reported a high rate (63.2%; 2005–2011) (National Cancer Institute, 2015). Early diagnosis and treatment lead to high OC survival rates in the United States. For OC, the American Cancer Society recommends cancer-related screenings every three years for people aged 20 to 39 years and annually for all people over 40 years old [35]. Poor prognosis is primarily due to the late detection of OC. Several imaging devices and technologies can help clinicians visualise and describe oral potentially malignant diseases in real time at the point of care [36,37,38]. These devices can detect changes in optical properties (i.e., absorption, scattering and fluorescence), which are related to precancerous cancer and cancer development and progression in oral tissue. In particular, the latest technological advances in optical and electronic components (e.g., cameras, tablets, high-power LED light sources, other consumer electronic products) have led to the development of a new generation of low-cost, portable visualization technology, which can be used as an auxiliary tool for assessing oral risk tissues/lesions.

The present study inevitably has several limitations. Firstly, the accuracy of the results relied on the quality and quantity of data in GBD 2019. Secondly, each etiology can only be analysed independently, thereby lacking data on the weight and synergistic effect of each etiology. Thirdly, data on other causes of OC, such as HPV infection, were not found and analysed.

## 5. Conclusions

The increasing incidence, mortality and DALY of OC pose a major public health challenge in the countries discussed above. The significant increase in the incidence of OC among people over 50 years old implies the need for increased OC screening among the elderly. Morbidity and mortality rates among male subjects are higher than among women. Government cancer programs are often expensive to run, especially in countries with large populations. Policy makers thus need to take these differences into account during policy formulation. Considering that Russia is a high-income economy with low survival rates, the issue of funding cancer control requires urgent attention. Control over SLT use in India, Bangladesh and Pakistan, which have low-middle SDI and high incidence rates, is critical.

## Figures and Tables

**Table 1:** The incident cases and age-standardized incidence rate of oral cancer in 1990 and 2019, and its temporal trends from 1990 to 2019. (Countries are ranked from highest to lowest in SDI.)

Nation	Sex	Incident Cases No.*102 (95%UI)		Change in Absolute Number (%)	ASIR per 100,000 No. (95%UI)		1990–2019 EAPC No. (95%CI)
		1990	2019		1990	2019	
Japan	both	3069.67 (2951.51–3164.97)	6945.3 (5688.46–8073.13)	126.26	1.85 (1.78–1.91)	2.24 (1.88–2.61)	0.68 (from 0.4 to 0.95)
USA	both	21,506.15 (20,863.25–22,016.96)	30,767.8 (26,450.35–35,861.98)	43.07	7.08 (6.88–7.24)	5.82 (5–6.8)	−0.77 (from −0.88 to −0.65)
Russian	both	5321.7 (5158.93–5530.02)	7369.72 (6377.52–8485.26)	38.48	2.94 (2.85–3.06)	3.29 (2.84–3.79)	0.07 (from −0.24 to 0.38)
China	both	12,390.24 (10,867.31–14,056.85)	45,216.41 (37,690.41–54,179.36)	264.94	1.4 (1.23–1.57)	2.25 (1.89–2.68)	2.33 (from 2.02 to 2.64)
Indonesia	both	2870.26 (2411.22–3416.52)	7305.53 (5315.64–9875.54)	154.53	2.72 (2.28–3.18)	3.38 (2.48–4.55)	0.68 (from 0.65 to 0.72)
Mexico	both	708.43 (686.68–724.99)	1913.03 (1647.8–2194.88)	170.04	1.62 (1.56–1.67)	1.63 (1.41–1.87)	−0.18 (from −0.28 to −0.07)
Brazil	both	3898.95 (3761.12–4027.84)	9582.82 (8998.64–100,77.52)	145.78	4.16 (3.99–4.3)	3.97 (3.73–4.18)	−0.18 (from −0.34 to −0.02)
India	both	39,064.72 (34,407.2–44,178.57)	104,838 (86,183.93–124,704.23)	168.37	7.93 (6.94–9.02)	8.82 (7.22–10.44)	0.23 (from 0.12 to 0.33)
Bangladesh	both	4244.93 (3096.55–5470.38)	8217.35 (5596.69–11,584.18)	93.58	8.37 (6.09–10.86)	6.12 (4.19–8.55)	−1.2 (from −1.36 to −1.05)
Pakistan	both	10,225.93 (8702.81–12,031.65)	28,579.23 (22,906.63–35,934.93)	179.48	16.6 (14.02–19.55)	21.93 (17.83–27.56)	0.85 (from 0.66 to 1.04)

**Table 2:** The death cases and age-standardized death rate of oral cancer in 1990 and 2019, and its temporal trends from 1990 to 2019. (Countries are ranked from highest to lowest in SDI.)

Nation	Sex	Death Cases No.*102 (95%UI)		Change in Absolute Number(%)	ASMR per 100,000 No. (95%UI)		1990–2019 EAPC No. (95%CI)
		1990	2019		1990	2019	
Japan	both	1771.13 (1686.56–1818.07)	4677.72 (3885.97–5115.54)	164.11	1.08 (1.03–1.11)	1.22 (1.07–1.3)	0.26 (from −0.04 to 0.57)
USA	both	5968 (5729.59–6122.36)	7821.12 (7373.7–8132.81)	31.05	1.92 (1.85–1.97)	1.4 (1.32–1.45)	−1.14 (from −1.31 to −0.97)
Russian	both	4502.4 (4358.54–4662.54)	5853.09 (4950.58–6852.39)	30.00	2.51 (2.43–2.61)	2.57 (2.18–3.01)	−0.32 (from −0.65 to 0.01)
China	both	7403.22 (6437.56–8357.89)	22,641.75 (18,908.09–27,077)	205.84	0.92 (0.81–1.03)	1.16 (0.98–1.38)	1.43 (from 1.14 to 1.73)
Indonesia	both	1717.05 (1437.36–1989.34)	4203.71 (3115.71–5690.08)	144.82	1.82 (1.52–2.09)	2.15 (1.59–2.9)	0.57 (from 0.52 to 0.61)
Mexico	both	457.42 (439.65–469.14)	1109 (957.6–1263.61)	142.45	1.14 (1.08–1.17)	0.98 (0.84–1.11)	−0.67 (from −0.78 to −0.56)
Brazil	both	2428.36 (2332.38–2512.56)	5563.6 (5201.97–5882.51)	129.11	2.76 (2.62–2.86)	2.34 (2.18–2.48)	−0.54 (from −0.68 to −0.41)
India	both	26,609.06 (23,261.73–30,578.45)	65,571.05 (54,391.75–78,443.01)	146.42	5.9 (5.14–6.78)	5.81 (4.84–6.94)	−0.22 (from −0.32 to −0.11)
Bangladesh	both	3032.04 (2203.98–3934.21)	5186.07 (3579.12–7217.8)	71.04	6.37 (4.63–8.2)	4.06 (2.83–5.6)	−1.67 (from −1.81 to −1.53)
Pakistan	both	7141.28 (6091.77–8406.48)	17,566.52 (14,062.12–22,168.62)	145.99	12.18 (10.34–14.32)	14.72 (11.9–18.34)	0.52 (from 0.29 to 0.74)

**Table 3:** The DALY and age-standardized DALY rate of Oral cancer in 1990 and 2019, and its temporal trends from 1990 to 2019. (Countries are ranked from highest to lowest in SDI.)

Nation	Sex	DALY No.*102 (95%UI)		Change in Absolute Number (%)	Age-Standardized DALY Rate per 100,000 No. (95%UI)		1990–2019 EAPC No. (95%CI)
		1990	2019		1990	2019	
Japan	Both	43,566.76 (42,191.13–44,497.11)	77,039.22 (68,375–81,994.44)	76.83	26.15 (25.32–26.71)	27.73 (25.76–29.06)	−0.01 (from −0.36 to 0.33)
USA	Both	150,227.05 (145,568.37–154,556.87)	180,924.75 (172,524.53–188,143.83)	20.43	51.04 (49.55–52.48)	35.02 (33.42–36.44)	−1.36 (from −1.54 to −1.19)
Russian	Both	130,291.33 (125,473.55–135,933.69)	163,106.13 (136,536.75–192,130.02)	25.19	71.78 (69.1–74.96)	74.92 (62.72–88.3)	−0.26 (from −0.62 to 0.11)
China	Both	222,647.1 (192,534.07–252,402.8)	575,805.4 (479,521.98–690,743.25)	158.62	23.63 (20.48–26.66)	28.27 (23.59–33.71)	1.24 (from 0.96 to 1.52)
Indonesia	Both	51,800.63 (43,542.12–60,910.65)	113,143.08 (83,375.61–153,308.74)	118.42	45.54 (38.08–52.9)	48.92 (36.22–66.05)	0.24 (from 0.19 to 0.29)
Mexico	Both	11,956.54 (11,666.31–12,191.73)	27,215.25 (23,357.09–31,173.64)	127.62	25.54 (24.78–26.14)	22.56 (19.36–25.77)	−0.56 (from −0.67 to −0.45)
Brazil	Both	71,379.4 (68,985.46–73,775.73)	148,871.92 (141,013.9–157,204.72)	108.56	72.01 (69.33–74.39)	60.82 (57.54–64.22)	−0.61 (from −0.79 to −0.44)
India	Both	847,614.72 (745,309.09–961,913.07)	1,922,663.79 (158,5385.47–2,318,025.04)	126.83	156.3 (137.01–178.41)	154.9 (128.07–186.18)	−0.13 (from −0.22 to −0.05)
Bangladesh	Both	93,543.29 (68,249.51–121,318.94)	144,651.02 (98,686.77–204,783.04)	54.64	171.71 (124.61–224.07)	103.95 (71.3–146.15)	−1.81 (from −1.93 to −1.68)
Pakistan	Both	222,000.03 (188,451.19–262,435.79)	594,088.79 (471,317.46–754,897.27)	167.61	343.14 (293.41–405.54)	421.87 (338.19–535.02)	0.57 (from 0.32 to 0.81)

## Data Availability

Data used for this publication are available on the website of the Institute for Health Metrics and Evaluation [9] and can be browsed or downloaded with free access.

## Supplementary Materials

The following supporting information can be downloaded at: https://www.mdpi.com/article/10.3390/ijerph19020875/s1, Figure S1: The EAPC of oral cancer ASR from 1990 to 2019; Figure S2: The proportion of different ages in Oral Cancer incidence by years; Figure S3: The ratio of male to female ASMR among different age groups in 2019; Figure S4: Distribution of different ages in Oral Cancer death in ten countries in 2019; Figure S5: The proportion of different ages in Oral Cancer death by years; Figure S6: The ratio of male to female age standardized DALY rate among different age groups in 2019; Figure S7: Distribution of different ages in Oral Cancer DALY in ten countries in 2019; Figure S8: The SMIR of oral cancer in 1990 and 2019, by sex and country; Table S1: The incident cases and age-standardized incidence rate of Oral cancer in 1990 and 2019, and its temporal trends from 1990 to 2019 of both female and male sex; Table S2: The death cases and age-standardized mortility rate of Oral cancer in 1990 and 2019, and its temporal trends from 1990 to 2019 of both female and male sex; Table S3: The DALY cases and age-standardized DALY rate of Oral cancer in 1990 and 2019, and its temporal trends from 1990 to 2019 of both female and male sex.
