# Supplementary material for: Burden of Oral Cancer on the 10 Most Populous Countries from 1990 to 2019: Estimates from the Global Burden of Disease Study 2019
Source: Int J Environ Res Public Health. 2022 Jan 13;19(2):875. doi: 10.3390/ijerph19020875 (PMC8775770; doi:10.3390/ijerph19020875)
Supplement: Supplementary file 1 [file ijerph-19-00875-s001.zip › ijerph-1523115-supplementary.pdf]

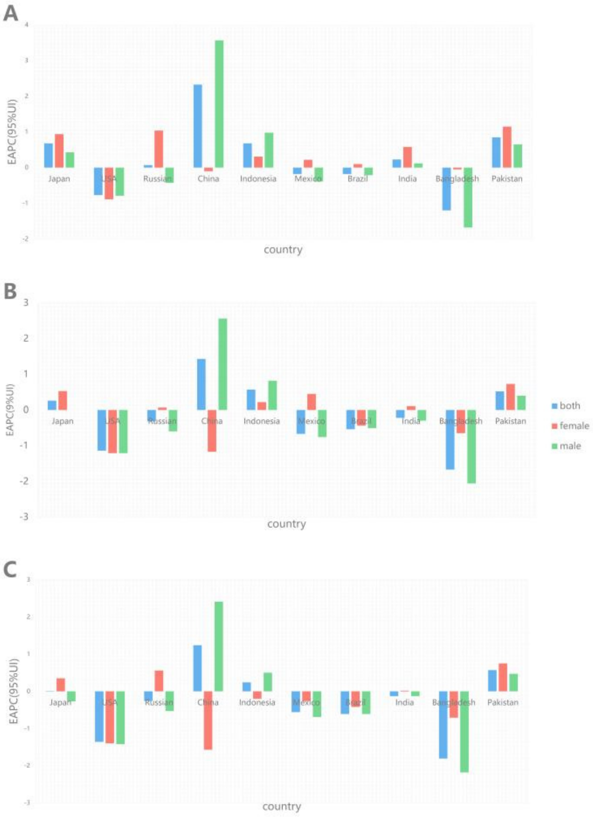

Figure S1 The EAPC of oral cancer ASR from 1990 to 2019, by sex and country. A: The EAPC of ASIR; B: The EAPC of ASMR; C: The EAPC of age-standardized DALY rate.

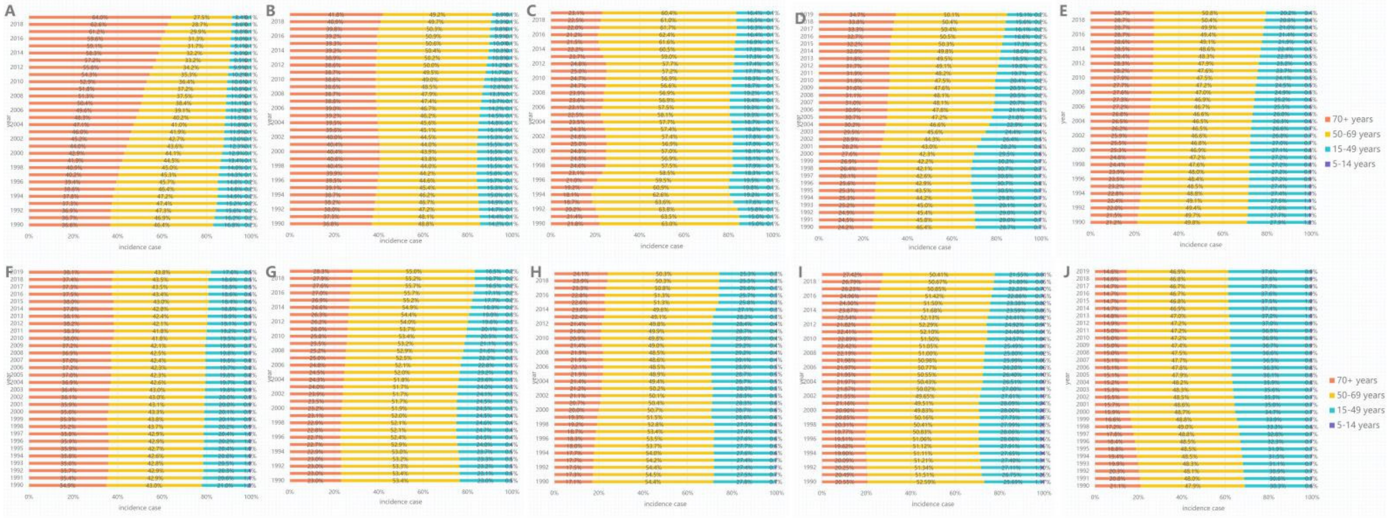

Figure S2 The proportion of different ages in Oral Cancer incidence by years. (A)Japan, (B)USA, (C)Russian, (D)China, (E)Indonesia, (F)Mexico, (G)Brazil, (H)India, (I)Bangladesh, (J)Pakistan. (Countries are ranked from highest to lowest in SDI)

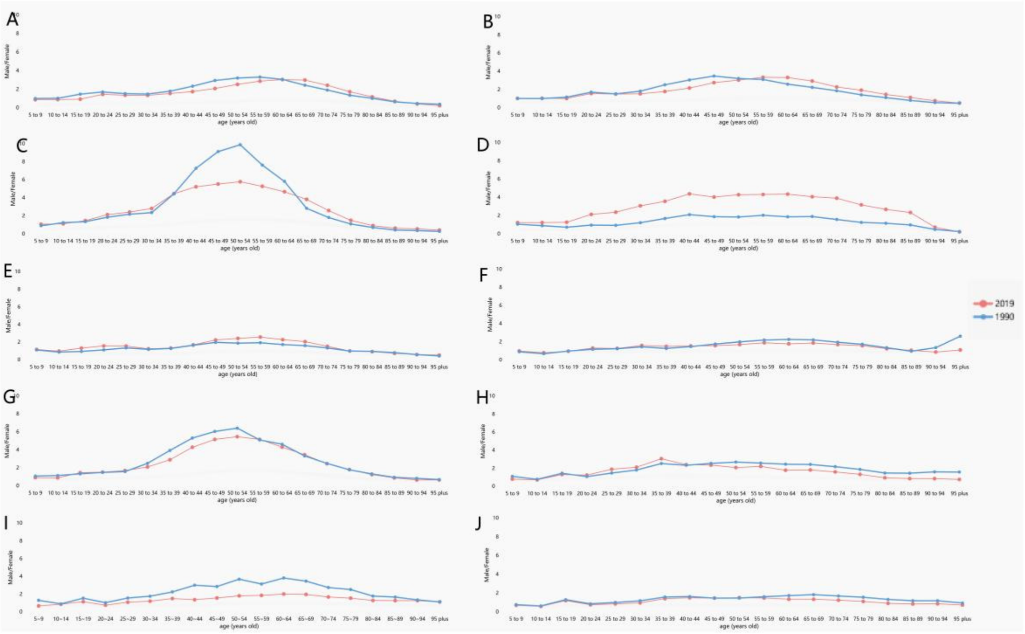

Figure S3 The ratio of male to female ASMR among different age groups in 2019, (A)Japan, (B)USA, (C)Russian, (D)China, (E)Indonesia, (F)Mexico, (G)Brazil, (H)India, (I)Bangladesh, (J)Pakistan. (Countries are ranked from highest to lowest in SDI)

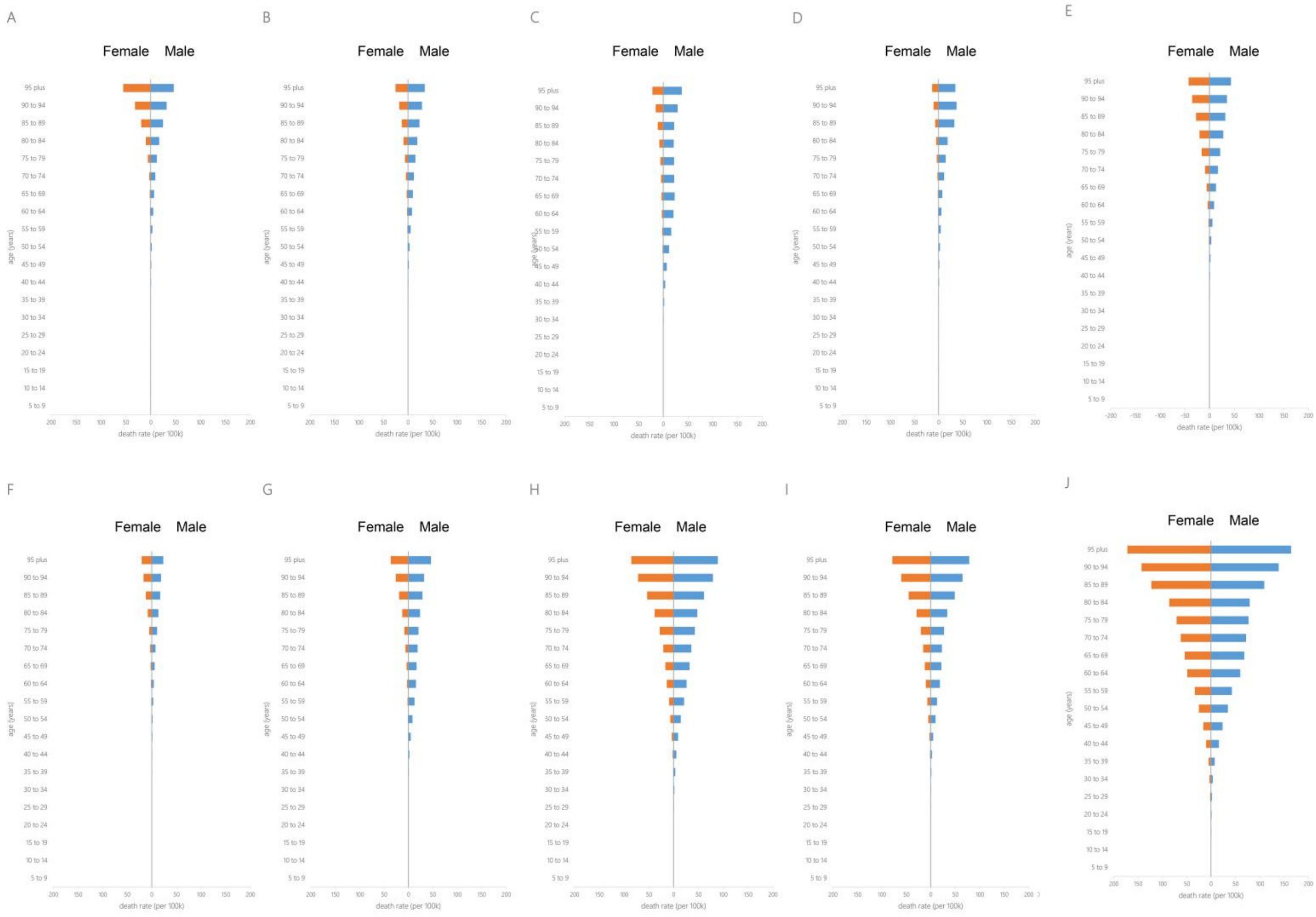

Figure S4 Distribution of different ages in Oral Cancer death in ten countries in 2019. (A)Japan, (B)USA, (C)Russian, (D)China, (E)Indonesia, (F)Mexico, (G)Brazil, (H)India, (I)Bangladesh, (J)Pakistan. (Countries are ranked from highest to lowest in SDI)

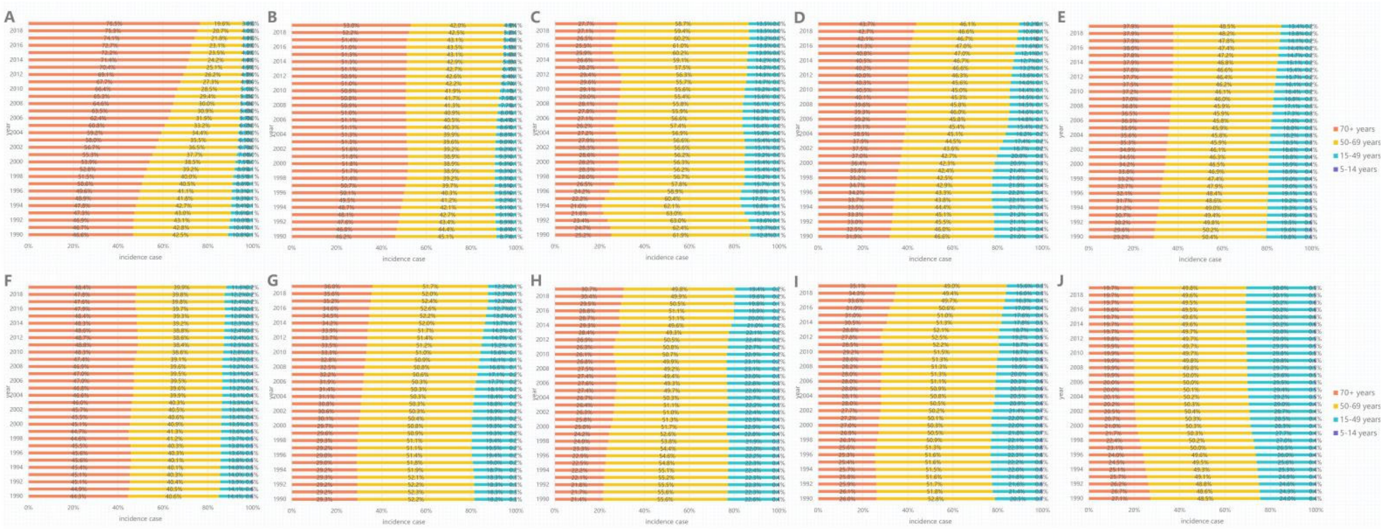

Figure S5 The proportion of different ages in Oral Cancer death by years. (A)Japan, (B)USA, (C)Russian, (D)China, (E)Indonesia, (F)Mexico, (G)Brazil, (H)India, (I)Bangladesh, (J)Pakistan. (Countries are ranked from highest to lowest in SDI)

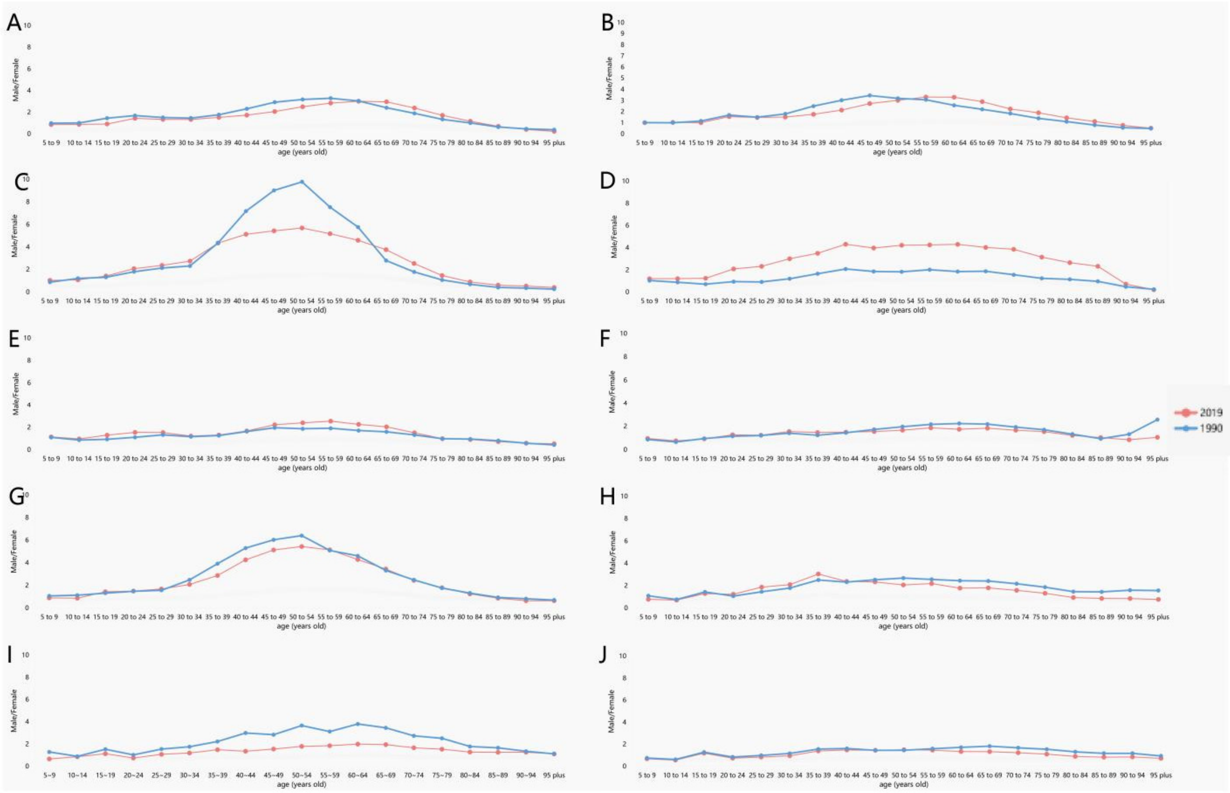

Figure S6. The ratio of male to female age standardized DALY rate among different age groups in 2019 (A)Japan, (B)USA, (C)Russian, (D)China, (E)Indonesia, (F)Mexico, (G)Brazil, (H)India, (I)Bangladesh, (J)Pakistan. (Countries are ranked from highest to lowest in SDI).

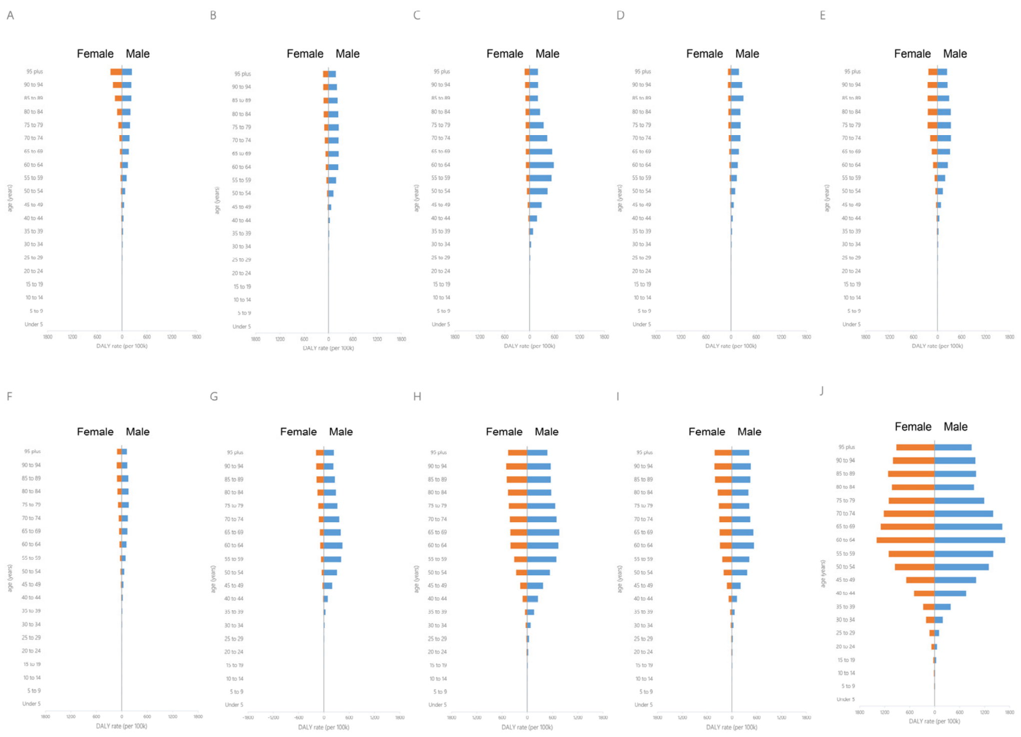

Figure S7 Distribution of different ages in Oral Cancer DALY in ten countries in 2019. (A)Japan, (B)USA, (C)Russian, (D)China, (E)Indonesia, (F)Mexico, (G)Brazil, (H)India, (I)Bangladesh, (J)Pakistan. (Countries are ranked from highest to lowest in SDI)

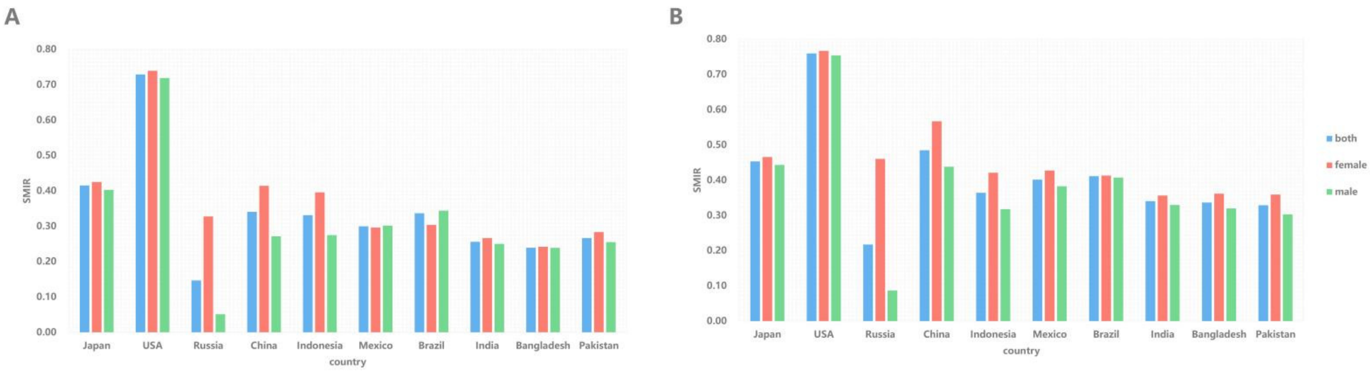

Figure S8 The SMIR of oral cancer in 1990 and 2019, by sex and country. A: The SMIR in 1990; B: The SMIR in 2019

Table S1. The incident cases and age-standardized incidence rate of Oral cancer in 1990 and 2019, and its temporal trends from 1990 to 2019 of both female and male sex. （Countries are ranked from highest to lowest in SDI）

Nation	Sex	Incident Cases No.*102 (95%UI)		Change in absolute number (%)	ASIR per 100,000 No.(95%UI)		1990-2019 EAPC No.(95%CI)
		1990	2019		1990	2019	

Japan	both	3069.67(2951.51-3164.97)	6945.3(5688.46-8073.13)	126.26	1.85(1.78-1.91)	2.24(1.88-2.61)	0.68(from 0.4 to 0.95)
USA	both	21506.15(20863.25-22016.96)	30767.8(26450.35-35861.98)	43.07	7.08(6.88-7.24)	5.82(5-6.8)	-0.77(from -0.88 to -0.65)
Russian	both	5321.7(5158.93-5530.02)	7369.72(6377.52-8485.26)	38.48	2.94(2.85-3.06)	3.29(2.84-3.79)	0.07(from -0.24 to 0.38)
China	both	12390.24(10867.31-14056.85)	45216.41(37690.41-54179.36)	264.94	1.4(1.23-1.57)	2.25(1.89-2.68)	2.33(from 2.02 to 2.64)
Indonesia	both	2870.26(2411.22-3416.52)	7305.53(5315.64-9875.54)	154.53	2.72(2.28-3.18)	3.38(2.48-4.55)	0.68(from 0.65 to 0.72)
Mexico	both	708.43(686.68-724.99)	1913.03(1647.8-2194.88)	170.04	1.62(1.56-1.67)	1.63(1.41-1.87)	-0.18(from -0.28 to -0.07)
Brazil	both	3898.95(3761.12-4027.84)	9582.82(8998.64-10077.52)	145.78	4.16(3.99-4.3)	3.97(3.73-4.18)	-0.18(from -0.34 to -0.02)
India	both	39064.72(34407.2-44178.57)	104838(86183.93-124704.23)	168.37	7.93(6.94-9.02)	8.82(7.22-10.44)	0.23(from 0.12 to 0.33)
Bangladesh	both	4244.93(3096.55-5470.38)	8217.35(5596.69-11584.18)	93.58	8.37(6.09-10.86)	6.12(4.19-8.55)	-1.2(from -1.36 to -1.05)
Pakistan	both	10225.93(8702.81-12031.65)	28579.23(22906.63-35934.93)	179.48	16.6(14.02-19.55)	21.93(17.83-27.56)	0.85(from 0.66 to 1.04)
Japan	female	1095.02(1018.7-1149.59)	6945.3(5688.46-8073.13)	167.95	1.2(1.12-1.26)	1.57(1.24-1.93)	0.94(from 0.67 to 1.21)
USA	female	7528.8(7169.63-7785.68)	30767.8(26450.35-35861.98)	30.94	4.3(4.12-4.43)	3.46(2.83-4.18)	-0.89(from -0.97 to -0.81)
Russian	female	1547.52(1465.18-1659.57)	7369.72(6377.52-8485.26)	60.57	1.38(1.3-1.49)	1.88(1.51-2.31)	1.04(from 0.86 to 1.22)
China	female	5334.84(4431.89-6296.44)	45216.41(37690.41-54179.36)	120.01	1.17(0.98-1.37)	1.16(0.94-1.43)	-0.1(from -0.19 to -0.01)
Indonesia	female	1312.63(999.51-1827.89)	7305.53(5315.64-9875.54)	134.59	2.43(1.88-3.28)	2.79(1.93-4.27)	0.31(from 0.24 to 0.39)

Mexico	female	268.32(257.93-276.6)	1913.03(1647.8-2194.88)	195.36	1.17(1.12-1.21)	1.26(1.05-1.52)	0.22(from 0.14 to 0.3)
Brazil	female	898.72(854.25-940.86)	9582.82(8998.64-10077.52)	179.17	1.91(1.8-2)	1.94(1.77-2.1)	0.1(from -0.07 to 0.28)
India	female	12347.23(10051.34-14842.43)	104838(86183.93-124704.23)	219.43	5.26(4.25-6.37)	6.64(5.27-8.16)	0.58(from 0.42 to 0.74)
Bangladesh	female	1155.04(873.67-1414.88)	8217.35(5596.69-11584.18)	186.04	4.95(3.76-6.19)	5.05(3.32-6.78)	-0.05(from -0.18 to 0.09)
Pakistan	female	4180.45(3391.87-5156.05)	28579.23(22906.63-35934.93)	216.45	14.74(11.8-18.27)	20.86(16.04-27.08)	1.15(from 1.01 to 1.28)
Japan	male	1974.65(1906.91-2043.23)	4011.24(3264.06-4928.74)	103.14	2.64(2.54-2.73)	2.96(2.4-3.64)	0.43(from 0.16 to 0.7)
USA	male	13977.35(13555.43-14380.2)	20909.48(16969.32-25665.82)	49.60	10.45(10.14-10.76)	8.48(6.89-10.39)	-0.79(from -0.94 to -0.65)
Russian Federation	male	3774.18(3585.06-3926.09)	4884.83(3966.59-5965.48)	29.43	5.43(5.18-5.64)	5.26(4.28-6.41)	-0.43(from -0.77 to -0.09)
China	male	7055.4(5737.35-8424.64)	33479.36(26151.96-42010.53)	374.52	1.68(1.38-1.99)	3.48(2.75-4.31)	3.57(from 3.13 to 4)
Indonesia	male	1557.63(1262.07-1861.04)	4226.19(2985.72-5820.03)	171.32	3.04(2.48-3.59)	4(2.87-5.42)	0.98(from 0.92 to 1.04)
Mexico	male	440.11(426.68-451.81)	1120.51(908.02-1365.53)	154.60	2.11(2.03-2.17)	2.05(1.67-2.49)	-0.39(from -0.53 to -0.26)
Brazil	male	3000.23(2879.03-3116.15)	7073.89(6578.88-7544.78)	135.78	6.65(6.37-6.91)	6.34(5.89-6.76)	-0.21(from -0.38 to -0.05)
India	male	26717.5(21949.2-32361.38)	65397.53(50291.98-81829.01)	144.77	10.41(8.43-12.6)	11.04(8.53-13.77)	0.12(from 0.02 to 0.21)
Bangladesh	male	3089.89(2031.24-4284.06)	4913.42(3348.71-7675.64)	59.02	11.18(7.43-15.42)	7.12(4.89-11.12)	-1.68(from -1.86 to -1.5)

Pakistan	male	6045.48(5059.22-7236.81)	15350.01(10953.73-21259.92)	153.91	18.16(15.13-21.86)	22.91(16.52-31.61)	0.65(from 0.42 to 0.88)

Table S2. The death cases and age-standardized death rate of oral cancer in 1990 and 2019, and its temporal trends from 1990 to 2019 of both female and male sex. （Countries are ranked from highest to lowest in SDI）

Nation	Sex	Death Cases No.*102 (95%UI)		Change in absolute number(%)	ASMR per 100,000 No.(95%UI)		1990-2019 EAPC No.(95%CI)
		1990	2019		1990	2019	
Japan	both	1771.13(1686.56-1818.07)	4677.72(3885.97-5115.54)	164.11	1.08(1.03-1.11)	1.22(1.07-1.3)	0.26(from -0.04 to 0.57)
USA	both	5968(5729.59-6122.36)	7821.12(7373.7-8132.81)	31.05	1.92(1.85-1.97)	1.4(1.32-1.45)	-1.14(from -1.31 to -0.97)
Russian	both	4502.4(4358.54-4662.54)	5853.09(4950.58-6852.39)	30.00	2.51(2.43-2.61)	2.57(2.18-3.01)	-0.32(from -0.65 to 0.01)
China	both	7403.22(6437.56-8357.89)	22641.75(18908.09-27077)	205.84	0.92(0.81-1.03)	1.16(0.98-1.38)	1.43(from 1.14 to 1.73)
Indonesia	both	1717.05(1437.36-1989.34)	4203.71(3115.71-5690.08)	144.82	1.82(1.52-2.09)	2.15(1.59-2.9)	0.57(from 0.52 to 0.61)
Mexico	both	457.42(439.65-469.14)	1109(957.6-1263.61)	142.45	1.14(1.08-1.17)	0.98(0.84-1.11)	-0.67(from -0.78 to -0.56)
Brazil	both	2428.36(2332.38-2512.56)	5563.6(5201.97-5882.51)	129.11	2.76(2.62-2.86)	2.34(2.18-2.48)	-0.54(from -0.68 to -0.41)
India	both	26609.06(23261.73-30578.45)	65571.05(54391.75-78443.01)	146.42	5.9(5.14-6.78)	5.81(4.84-6.94)	-0.22(from -0.32 to -0.11)
Bangladesh	both	3032.04(2203.98-3934.21)	5186.07(3579.12-7217.8)	71.04	6.37(4.63-8.2)	4.06(2.83-5.6)	-1.67(from -1.81 to -1.53)
Pakistan	both	7141.28(6091.77-8406.48)	17566.52(14062.12-22168.62)	145.99	12.18(10.34-14.32)	14.72(11.9-18.34)	0.52(from 0.29 to 0.74)
Japan	female	638.02(586.43-664.32)	2160.01(1630.33-2491.74)	238.55	0.69(0.63-0.72)	0.84(0.69-0.94)	0.53(from 0.23 to 0.83)

USA	female	2088.37(1949.67-2169.57)	2579.37(2324.19-2743.26)	23.51	1.12(1.06-1.16)	0.81(0.74-0.85)	-1.21(from -1.32 to -1.1)
Russian	female	1053.08(997.25-1120.77)	1447.36(1201.62-1733.88)	37.44	0.93(0.88-0.99)	1.01(0.84-1.22)	0.07(from -0.13 to 0.27)
China	female	2832.55(2355.39-3331.1)	5033.76(4058.52-6116.89)	77.71	0.69(0.57-0.8)	0.5(0.41-0.61)	-1.17(from -1.23 to -1.11)
Indonesia	female	693.04(534.67-932.59)	1592.12(1082.05-2477.44)	129.73	1.47(1.16-1.91)	1.62(1.11-2.45)	0.22(from 0.11 to 0.33)
Mexico	female	168.81(161.12-174.08)	439.29(365.6-528.77)	160.23	0.83(0.78-0.86)	0.72(0.6-0.87)	-0.45(from -0.54 to -0.35)
Brazil	female	571.58(537.79-598.77)	1475.74(1325.37-1598.41)	158.19	1.33(1.23-1.4)	1.14(1.02-1.23)	-0.44(from -0.57 to -0.32)
India	female	8084.35(6537.11-9732.17)	23941.6(19153-29543.76)	196.15	3.86(3.09-4.69)	4.27(3.41-5.25)	0.11(from -0.05 to 0.27)
Bangladesh	female	788.72(595.94-975.98)	1957.43(1308.98-2632.2)	148.18	3.75(2.81-4.72)	3.22(2.13-4.32)	-0.65(from -0.75 to -0.55)
Pakistan	female	2804.13(2265.27-3463.66)	7611.25(5829.23-9903.6)	171.43	10.56(8.41-13.1)	13.37(10.43-17.04)	0.73(from 0.57 to 0.89)
Japan	male	1133.11(1099.13-1157.71)	2517.71(2254.3-2667.46)	122.19	1.58(1.52-1.61)	0.84(0.69-0.94)	0(from -0.31 to 0.3)
USA	male	3879.63(3762.75-3982.09)	5241.75(4977.76-5448.65)	35.11	2.94(2.84-3.01)	0.81(0.74-0.85)	-1.21(from -1.42 to -1)
Russian	male	3449.32(3284.25-3585.01)	4405.73(3580.59-5367.56)	27.73	5.15(4.93-5.35)	1.01(0.84-1.22)	-0.6(from -0.94 to -0.26)
China	male	4570.67(3724.24-5446.74)	17607.99(14093.63-21897.35)	285.24	1.23(1.02-1.44)	0.5(0.41-0.61)	2.56(from 2.17 to 2.96)
Indonesia	male	1024.01(828.7-1217.39)	2611.59(1899.17-3588.03)	155.04	2.2(1.78-2.59)	1.62(1.11-2.45)	0.82(from 0.77 to 0.88)
Mexico	male	288.61(277.81-296.8)	669.71(552.32-813.23)	132.05	1.47(1.41-1.52)	0.72(0.6-0.87)	-0.76(from -0.89 to -0.63)

Brazil	male	1856.78(1780.24-1927.32)	4087.86(3810.01-4351.2)	120.16	4.36(4.16-4.54)	1.14(1.02-1.23)	-0.51(from -0.65 to -0.37)
India	male	18524.71(14975.06-22440.18)	41629.45(32101.61-52775.27)	124.72	7.81(6.19-9.48)	4.27(3.41-5.25)	-0.3(from -0.4 to -0.21)
Bangladesh	male	2243.31(1491.87-3067.97)	3228.65(2214.08-4972.22)	43.92	8.51(5.71-11.56)	3.22(2.13-4.32)	-2.06(from -2.23 to -1.88)
Pakistan	male	4337.15(3634.25-5263.81)	9955.27(7044.3-13628.43)	129.53	13.53(11.24-16.4)	13.37(10.43-17.04)	0.4(from 0.13 to 0.67)

Table S3. The DALY and age-standardized DALY rate of Oral cancer in 1990 and 2019, and its temporal trends from 1990 to 2019 of both female and male sex. （Countries are ranked from highest to lowest in SDI）

		DALY No.*102 (95%UI)		Change in absolute number(%)	Age-standardized DALY rate per 100,000 No.(95%UI)		1990-2019 EAPC No.(95%CI)
Nation	Sex	1990	2019		1990	2019	
Japan	Both	43566.76(42191.13-44497.11)	77039.22(68375-81994.44)	76.83	26.15(25.32-26.71)	27.73(25.76-29.06)	-0.01(from -0.36 to 0.33)
USA	Both	150227.05(145568.37-154556.87)	180924.75(172524.53-188143.83)	20.43	51.04(49.55-52.48)	35.02(33.42-36.44)	-1.36(from -1.54 to -1.19)
Russian	Both	130291.33(125473.55-135933.69)	163106.13(136536.75-192130.02)	25.19	71.78(69.1-74.96)	74.92(62.72-88.3)	-0.26(from -0.62 to 0.11)
China	Both	222647.1(192534.07-252402.8)	575805.4(479521.98-690743.25)	158.62	23.63(20.48-26.66)	28.27(23.59-33.71)	1.24(from 0.96 to 1.52)
Indonesia	Both	51800.63(43542.12-60910.65)	113143.08(83375.61-153308.74)	118.42	45.54(38.08-52.9)	48.92(36.22-66.05)	0.24(from 0.19 to 0.29)
Mexico	Both	11956.54(11666.31-12191.73)	27215.25(23357.09-31173.64)	127.62	25.54(24.78-26.14)	22.56(19.36-25.77)	-0.56(from -0.67 to -0.45)
Brazil	Both	71379.4(68985.46-73775.73)	148871.92(141013.9-157204.72)	108.56	72.01(69.33-74.39)	60.82(57.54-64.22)	-0.61(from -0.79 to -0.44)

India	Both	847614.72(745309.09-961913.07)	1922663.79(1585385.47-2318025.04)	126.83	156.3(137.01-178.41)	154.9(128.07-186.18)	-0.13(from -0.22 to -0.05)
Bangladesh	Both	93543.29(68249.51-121318.94)	144651.02(98686.77-204783.04)	54.64	171.71(124.61-224.07)	103.95(71.3-146.15)	-1.81(from -1.93 to -1.68)
Pakistan	Both	222000.03(188451.19-262435.79)	594088.79(471317.46-754897.27)	167.61	343.14(293.41-405.54)	421.87(338.19-535.02)	0.57(from 0.32 to 0.81)
Japan	Female	13668.67(13016.54-14088.09)	29029.42(23711.61-32528.47)	112.38	15.28(14.59-15.72)	17.78(15.95-19.46)	0.35(from 0.02 to 0.68)
USA	Female	46753.13(44617-48458.5)	52851.08(49658.03-55903.3)	13.04	27.94(26.86-28.87)	19.13(18.15-20.12)	-1.4(from -1.53 to -1.27)
Russian	Female	23966.2(22724.58-25831.66)	33791.1(27777.77-41027.18)	40.99	21.87(20.7-23.72)	26.58(21.62-32.62)	0.56(from 0.32 to 0.8)
China	Female	84052.37(69025.32-99392.04)	121020.61(97701.64-147266.99)	43.98	17.75(14.66-21.05)	11.94(9.65-14.51)	-1.57(from -1.66 to -1.48)
Mexico	Female	4369.02(4239.11-4484.04)	10448.97(8652.05-12690.26)	139.16	17.76(17.15-18.27)	16.33(13.59-19.78)	-0.26(from -0.35 to -0.18)
Indonesia	Female	20295.46(15398.02-28495.56)	39956.41(27019.42-62841.9)	96.87	35.12(26.98-48)	34.58(23.6-53.53)	-0.2(from -0.31 to -0.08)
Brazil	Female	14597.72(13979.15-15241.79)	33144.63(30435-35625.78)	127.05	29.17(27.76-30.5)	25.54(23.45-27.46)	-0.42(from -0.59 to -0.25)
India	Female	256991.22(210436.88-307275.64)	657254.15(525800.86-814995.7)	155.75	99.46(80.51-119.81)	106.8(85.53-132.12)	0.02(from -0.15 to 0.18)
Bangladesh	Female	24631.94(18022.86-30290.27)	54708.58(36661.54-74089.54)	122.10	96.05(72.43-117.42)	79.97(53.63-107.77)	-0.71(from -0.83 to -0.59)
Pakistan	Female	88319.22(71614.52-108103.48)	256585.9(193335.05-338597.53)	190.52	293.08(236.28-360.39)	375.37(286.27-490.06)	0.75(from 0.58 to 0.93)
Japan	Male	29898.09(29223.96-30511.44)	48009.79(44504.04-50301.98)	60.58	38.79(37.85-39.58)	38.29(35.96-39.96)	-0.27(from -0.61 to 0.08)

USA	Male	103473.92(100302.5-106781.87)	128073.66(121372.63-133885.28)	23.77	78.21(75.84-80.63)	52.6(49.95-54.95)	-1.42(from -1.62 to -1.22)
Russian	Male	106325.13(100292.3-111022.66)	129315.02(104665.16-158228.85)	21.62	143.84(136.23-150.05)	137.99(111.98-168.74)	-0.53(from -0.9 to -0.15)
China	Male	138594.73(112345.93-166477.25)	454784.79(360260.7-571484.69)	228.14	29.96(24.49-35.71)	45.75(36.55-56.94)	2.41(from 2.02 to 2.81)
Indonesia	Male	31505.16(25412.52-37920.42)	73186.66(52210.72-100887.05)	132.30	56.56(45.85-67.24)	64.03(46.64-87.82)	0.5(from 0.45 to 0.55)
Mexico	Male	7587.52(7394.87-7774.53)	16766.28(13753.98-20416.68)	120.97	33.95(32.93-34.88)	29.66(24.41-36.13)	-0.69(from -0.83 to -0.56)
Brazil	Male	56781.68(54581.72-58989.15)	115727.3(108453.59-123293.81)	103.81	118.74(114.01-123.43)	101.09(94.67-107.7)	-0.61(from -0.79 to 0.42)
India	Male	590623.5(485181.29-711151.88)	1265409.64(969182.49-1605569.46)	114.25	208.51(168.72-252.02)	203.16(156.31-257)	-0.13(from -0.22 to -0.03)
Bangladesh	Male	68911.35(44869.04-95644.93)	89942.43(60755.41-140800.16)	30.52	234.52(154.29-323.89)	127.11(86.41-197.85)	-2.18(from -2.33 to -2.03)
Pakistan	Male	133680.81(111855.2-161308.95)	337502.89(238392.46-464856.86)	152.47	386.4(324.32-467.59)	465.64(329.94-635.07)	0.47(from 0.17 to 0.76)
